# Psychiatric manifestations of paraneoplastic syndromes

**DOI:** 10.1192/j.eurpsy.2022.1698

**Published:** 2022-09-01

**Authors:** M. Lemos, A. Lourenço, M. Ribeiro

**Affiliations:** Centro Hospitalar Lisboa Norte, Psychiatry, Lisboa, Portugal

**Keywords:** endrocrine paraneoplastic syndromes, neuropsychiatric manifestations, limbic encephalitis, paraneoplastic syndromes

## Abstract

**Introduction:**

Paraneoplastic syndromes (PS) result from indirect effects of neoplasms. In 50% of the cases the symptoms precede the diagnosis and run independently. PS may involve the peripheral or central nervous system, resulting in symptoms from sensory neuropathies to several neuropsychiatric manifestations.

**Objectives:**

To review the psychiatric manifestations of paraneoplastic syndromes affecting the nervous system.

**Methods:**

Selective literature review via PubMed search, using the keywords “paraneoplastic syndromes”, “endrocrine paraneoplastic syndromes”, “neuropsychiatric manifestations”, “limbic encephalitis”.

**Results:**

The prevalence of PS varies with the type of cancer (<1% for breast and ovarian cancers; 3-5% for small cell lung cancer; 20% for thymomas). The general mechanisms behind PS are related to the production of substances by the tumor that directly or indirectly cause distant symptoms, the depletion of substances or the host response to the tumor. Frequently there are autoimmune phenomena involved, with the production of antineuronal antibodies that recognise various antigens at the nervous system. Paraneoplastic neurological disorders include limbic encephalits that can present subacutely with symptoms of depression, irritability, hallucinations, cognitive impairment associated with sleep alterations, confusion and seizures. Others include psoclonus-myoclonus ataxia syndrome, neuromyotonia and cramp fasciculation syndrome. Metabolic and endocrine paraneoplastic syndromes (hypercortisolism, carcinoid tumors, pancreatic cancer) can result from the production of cytokines and hormones by the tumor and produce mood disorders, confusional states and psychosis.

**Conclusions:**

PS can be related to various neuropsychiatric manifestations affecting consciousness, cognition, mood and perception. The recognition of this association can alert for the possibility of a cancer diagnosis specially when facing a patient with unusual clinical presentation.

**Disclosure:**

No significant relationships.

